# Neutrophil/lymphocyte ratio has a prognostic value for patients with terminal cancer

**DOI:** 10.1186/s12957-016-0904-7

**Published:** 2016-05-16

**Authors:** Yoichi Nakamura, Ryohei Watanabe, Miwa Katagiri, Yoshihisa Saida, Natsuya Katada, Manabu Watanabe, Yasushi Okamoto, Koji Asai, Toshiyuki Enomoto, Takaharu Kiribayashi, Shinya Kusachi

**Affiliations:** Palliative Care Team, Toho University Ohashi Medical Center, 2-17-6 Ohashi, Meguroku, Tokyo, 1538515 Japan; Department of Surgery, Toho University Ohashi Medical Center, Meguroku, Tokyo, Japan

**Keywords:** Blood cells, Inflammation, Neoplasms, Palliative care, Prognosis

## Abstract

**Background:**

Determining prognosis in advanced cancer is of key importance. Various prognostic scores have been developed. However, they are often very complex. In this study, we evaluated the feasibility of neutrophil/lymphocyte ratio (NLR) as an index to estimate survival in terminal cancer patients.

**Methods:**

NLR was calculated retrospectively based on blood tests performed at 3 months, 2 months, 4 weeks, 3 weeks, 2 weeks, 1 week, and within 3 days before death in 160 cancer patients (82 men, 78 women; age range, 33–99 years; mean age, 69.8 years).

**Results:**

NLR increased significantly with time (*P* < 0.0001). Mean NLR was significantly higher in patients who died within 4 weeks (29.82) than in those who lived more than 4 weeks (6.15). The NLR cutoff point was set at 9.21 according to receiver operating characteristic curve analysis (area under the curve, 0.82; 95 % confidence interval, 0.79–0.85). We inferred that life expectancy would be <4 weeks when NLR >9.21. The sensitivity, specificity, positive predictive value, and negative predictive value were 65.6, 84.1, 90.6, and 51.1 %, respectively. The positive and negative likelihood ratios were 4.125 and 0.409, respectively.

**Conclusions:**

NLR appears to be a useful and simple parameter to predict the clinical outcomes of patients with terminal cancer.

## Background

Determining prognosis in patients with advanced cancer is of key importance. Previous studies have shown that physicians do not accurately estimate survival time [1, 2]. A systematic review of the accuracy of clinical predictions of survival in terminally ill cancer patients has shown that physicians consistently overestimate survival; however, their predictions are highly correlated with actual survival [3].

Various prognostic scores have been developed. The palliative prognostic score (PPS) [4] and the palliative prognostic index (PPI) [5] are frequently used for prognostic estimates in patients with terminal-stage cancer, but these methods are not simple and often include subjective factors. These scores are widely used by palliative care physicians to make the remaining time that patients have left to live more meaningful. However, oncologists regard them as complex predictive formula. The inability to make accurate forecasts about a patient’s prognosis is a reason why treatment often runs counter to the patient’s expectations. Therefore, we consider it important to have an index that enables doctors to easily predict the prognosis of cancer patients.

Neutrophil/lymphocyte ratio (NLR) is an immunological index commonly used in cancer therapy and diagnosis [6–12]. In this study, we evaluated the feasibility of NLR as an index to estimate survival in terminal cancer patients.

## Methods

This retrospective study included data from 160 consecutive patients who received palliative care and died in the Toho University Ohashi Medical Center between May 2011 and November 2013. The inclusion criteria were (i) histologically or clinically confirmed malignant tumor and (ii) death after hospitalization. We implemented a comprehensive agreement method to obtain consent from the patients regarding the study. The study was approved by the ethical review board of Toho University Ohashi Medical Center.

Data on white blood cell (WBC) counts and neutrophil and lymphocyte fractions (%) were extracted retrospectively from electronic records of blood tests performed at 3 months, 2 months, 4 weeks, 3 weeks, 2 weeks, 1 week, and within 3 days before death of the cancer patients. NLR was calculated by dividing the absolute count of band and segmented neutrophils by the number of lymphocytes in the complete blood count.

All statistical analyses were performed with EZR (Saitama Medical Center, Jichi Medical University, Saitama, Japan), the graphical user interface for R (The R Foundation for Statistical Computing, Vienna, Austria). More precisely, EZR is a modified version of R commander designed to add statistical functions frequently used in biostatistics [13]. Continuous variables were expressed as mean ± standard deviation. Comparisons between time points were performed using the Friedman rank sum test. Comparisons between groups were performed using the *t* test. The receiver operating characteristic (ROC) curve was generated by plotting the sensitivity value against the false-positive rate (1-specificity). We assessed the predictive value of life expectancy <4 weeks by calculating the area under the curve (AUC) and estimated the optimal cutoff value based on maximum sensitivity and specificity. Differences were considered statistically significant if the null hypothesis could be rejected with >95 % confidence (*P* < 0.05).

## Results

The average age of the patients (82 men, 78 women) was 69.8 years (age range, 33–99 years). Patient characteristics and primary tumor sites are shown in Table 1. The feasibility of using NLR was evaluated from 735 data points.

**Table 1:** Patient characteristics (*n* = 160)

Age, mean (range)	69.8 (33–99) years			
Sex, male/female	82:78			
Site of primary tumor	Lung	33	CUP	7
	Colon/rectum	32	Esophagus	5
	Stomach	25	Cervix	5
	Breast	13	Uterine body	4
	Pancreas	11	Ovary	3
	Biliary tract	7	Ureter	2
	Bladder	7	Other	6

*CUP* cancer of unknown primary

**Fig. 1:**
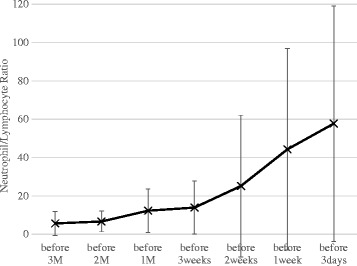
The time course of the NLR value. NLR values increased significantly with time

The median NLR values were 3.83 at 3 months before death, 5.38 at 2 months, 8.53 at 4 weeks, 10.22 at 3 weeks, 11.48 at 2 weeks, 21.16 at 1 week, and 37.40 within 3 days (Table 2). NLR values increased significantly with time (*P* < 0.0001) (Fig. 1).

**Table 2:** NLR values at various times before death

Time before death	Numbers of patients	Neutrophils × 10^9^/L, mean ± standard deviation	Lymphocytes × 10^9^/L, mean ± standard deviation	NLR, mean ± standard deviation (median)
3 months	109	4.84 ± 3.99	1.07 ± 0.54	5.64 ± 6.30 (3.83)
2 months	111	5.62 ± 3.45	0.99 ± 0.52	6.65 ± 5.36 (5.38)
4 weeks	112	7.93 ± 5.02	0.87 ± 0.49	12.28 ± 11.25 (8.53)
3 weeks	99	7.90 ± 4.23	0.78 ± 0.50	13.91 ± 13.83 (10.22)
2 weeks	110	9.10 ± 5.71	0.84 ± 1.03	25.15 ± 36.93 (11.48)
1 week	100	11.53 ± 7.48	0.55 ± 0.41	44.28 ± 56.62 (21.16)
3 days	96	13.50 ± 11.25	0.52 ± 0.71	57.74 ± 61.41 (37.40)

The mean NLR value of patients who lived more than 4 weeks was 6.15 ± 5.86, while that of those who died within 4 weeks was 29.82 ± 44.19; a significant difference was observed between the two groups (*P* < 0.00000001) (Table 3).

**Table 3:** Mean NLR of the patients who lived more than or up to 4 weeks

	More than 4 weeks	Up to 4 weeks	*P* value
NLR, mean ± standard deviation	6.15 ± 5.86	29.82 ± 44.19	*P* < 0.00000001

**Fig. 2:**
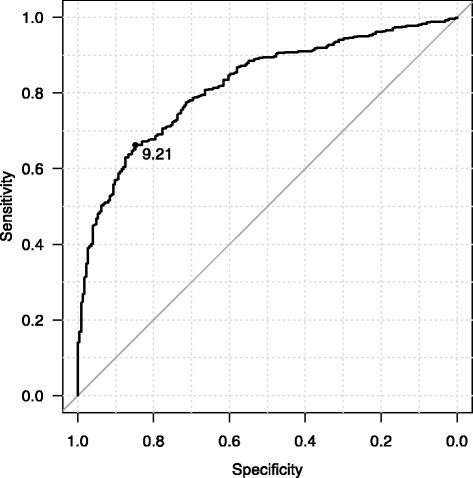
The ROC curve of NLR (lived more than or up 4 weeks). Receiver operating characteristic curve analysis of NLR in the patients who lived more than or up to 4 weeks. The cutoff point was set to 9.21. Area under the curve, 0.82; 95 % confidence interval, 0.79–0.85

In ROC analysis, the area under the curve was 0.82 (95 % confidence interval (CI), 0.79–0.85) (Fig. 2). Using a cutoff point of NLR of 9.21, life expectancy was assumed to be <4 weeks yielded a sensitivity of 65.6 %, (95 % CI, 61.4–69.7 %), specificity of 84.1 % (95 % CI, 78.6–88.7 %), positive predictive value (PPV) of 90.6 % (95 % CI, 87.2–93.4 %), negative predictive value (NPV) of 51.1 % (95 % CI, 45.8–56.4 %), positive likelihood ratio of 4.125 (95 % CI, 3.025–5.626), and negative likelihood ratio of 0.409 (95 % CI, 0.358–0.467).

## Discussion

Prognostic scoring tools are applied to predict the life expectancy of terminal cancer patients. In daily clinical practice, tools such as PPS and PPI are used by palliative care physicians [4, 5]. Here, we suggest the use of NLR as a simple tool to estimate survival in terminal cancer patients.

PPS grades the clinical prediction of survival, anorexia, Karnofsky’s performance status, dyspnea, WBC count, and lymphocyte count. A lower grade means a lower life expectancy (within 30 days). One disadvantage of this tool is that it considers the clinical prediction of survival, which is a subjective parameter [4].

PPI includes various parameters such as the existence of edema, dyspnea at rest, delirium, PPS grade, and the amount of oral intake. However, some of these parameters are also subjective. In patients with PPI grade 6 or higher, life expectancy is <3 weeks with a sensitivity of 83 %, specificity of 85 %, PPV of 80 %, and NPV of 87 %. In patients with PPI grade 4 or higher, the sensitivity is 79 %, specificity is 77 %, PPV is 83 %, and NPV is 71 % [5].

Besides their subjectivity, PPS and PPI are complex. Therefore, they are not commonly used by specialists other than palliative care physicians (e.g., oncologists and general practitioners). Accurately predicting life expectancy would enable clinicians to provide more appropriate end-of-life care for terminally ill patients, who would like to think how they wish to spend their remaining time. However, unreliable predictions could lead clinicians to administer unnecessary body-rending chemotherapy and mislead the patients’ preferences. These problems are more likely to occur in cases in which palliative care physicians are not involved.

According to Glare et al. [3], physicians tend to interpret the prognosis more optimistically. Therefore, easy-to-use, objective prognostic tools are needed. The development of such tools could be useful for oncologists and healthcare providers in daily clinical and home-based care.

In our earlier studies, we discussed about the prognostic nutritional index (Onodera’s PNI), which depends only on objective data such as serum albumin level and peripheral lymphocyte count [14]. This was originally used in digestive surgery to evaluate the patients’ preoperative nutritional condition, and anastomosis of digestive tract was considered contraindicated in cases of grades of 40 and under [15]. It was also considered to be a prognosis index in stage IV cancer of the digestive tract. It was used as a vague predictive formula that estimated the possibility of death within 60 days when the patient was graded 35 and under. PNI showed lower value as time proceeded, and therefore, it was considered a feasible factor to estimate the prognosis of patients with terminal cancer.

In this study, we evaluated NLR as a simple prognostic index of survival in terminal cancer patients. This method requires minimal blood test items, and thus, it would be a least invasive approach to the patient. The prognosis prediction by NLR is believed to have moderate accuracy, with 0.81 area under the curve according to ROC analysis and a 4.125 positive likelihood ratio.

It has been reported that critically ill cancer patients in the intensive care unit exhibit high NLR values [16]. Other studies have shown that pre-treatment NLR could serve as a feasible prognosis factor in cases of non-small-cell lung cancer [6], ureteral cancer [7], esophageal squamous cell cancer [8], biliary tract cancer [9], unresectable gastric cancer [10], liver cancer [11], and pancreatic cancer [12].

NLR is an inflammation marker. There is a strong relationship between inflammation and cancer. But the mechanism of neutrophilia in cancer patients is not fully understood, and it is believed to be the result of a combination of factors. One obvious cause of neutrophilia is paraneoplastic production of myeloid growth factors, such as granulocyte colony-stimulating factor, by cancer cells themselves. Other possible factors that cause neutrophilia are coexistent infection and cancer-related inflammation [17]. Lymphocytes play a critical role in tumor defense, inducing cytotoxic cell death through the immune response. Lymphopenia is frequently observed in patients with advanced cancer, reflecting cancer-related immunosuppression [18]. Therefore, neutrophilia and lymphopenia worsen with the progression of cancer. NLR increases with time in patient with terminal cancer.

This study also has some limitations. First, it was performed on a relatively small population, but our major aim in this pilot study was merely to assess the feasibility of using the NLR as an index to estimate survival in patients with terminal cancer, using our consecutive patients. Second, this study had a retrospective, single-center design, and a potential bias in the selection of patients. Finally, this study analyzed patients with various cancer, and it is not known whether the significance of the NLR for predicting life expectancy is consistent irrespective of the type of cancer. To improve the prediction of prognosis of terminal cancer, it may be necessary to combine other indices together with the NLR. Thus, larger, prospective studies will need to be performed to confirm these preliminary results.

## Conclusions

In the present study, the NLR has emerged as a feasible factor for prognosis prediction in terminal-stage cancer. NLR is easy to determine based on blood test results and could be applied by cancer clinicians as well as healthcare providers who do not specialize in palliative medicine.

### Availability of data and material section

Raw data https://zenodo.org/record/50485.

## Abbreviations

CI: confidence interval
CUP: cancer of the unknown primary
NLR: neutrophil/lymphocyte ratio
NPV: negative predictive value
PNI: prognostic nutritional index
PPI: palliative prognostic index
PPS: palliative prognostic score
PPV: positive predictive value
ROC: receiver operating characteristic
WBC: white blood cell

## References

[1] Vigano A, Dorgan M, Bruera E, Suaretz-Almazor ME (1999). The relative accuracy of clinical estimation of the duration of life for patients with end of life cancer. Cancer.

[2] Christakis NA, Lamont EB (2000). Extent and determinants of error in doctors’ prognosis in terminally ill patients: prospective cohort study. BMJ.

[3] Glare P, Virik K, Jones M, Hudson M, Eychmuller S, Simes J (2003). A systematic review of physicians’ survival predictions in terminally ill cancer patients. BMJ.

[4] Pirovano M, Maltoni M, Nanni O, Marinari M, Indelli M, Zaninetta G (1999). A new palliative prognostic score: a first step for the staging of terminally ill cancer patients. J Pain Symptom Manage.

[5] Morita T, Tsunoda J, Inoue S, Chihara S (1999). The palliative prognostic index: a scoring system for survival prediction of terminally ill cancer patients. Supprot Care Cancer.

[6] Kacan T, Babacan NA, Seker M, Yucel B, Bahceci A, Eren AA (2014). Could the neutrophil to lymphocyte ratio be a poor prognostic factor for non small cell lung cancers?. Asian Pac J Cancer Prev.

[7] Dalpiaz O, Pichler M, Mannweiler S, Martín Hernández JM, Stojakovic T, Pummer K (2014). Validation of the pretreatment derived neutrophil-lymphocyte ratio as a prognostic factor in a European cohort of patients with upper tract urothelial carcinoma. Br J Cancer.

[8] Feng JF, Huang Y, Chen QX (2014). Preoperative platelet lymphocyte ratio (PLR) is superior to neutrophil lymphocyte ratio (NLR) as a predictive factor in patients with esophageal squamous cell carcinoma. World J Surg Oncol.

[9] McNamara MG, Templeton AJ, Maganti M, Walter T, Horgan AM, McKeever L (2014). Neutrophil/lymphocyte ratio as a prognostic factor in biliary tract cancer. Eur J Cancer.

[10] Li QQ, Lu ZH, Yang L, Lu M, Zhang XT, Li J (2014). Neutrophil count and the inflammation-based glasgow prognostic score predict survival in patients with advanced gastric cancer receiving first-line chemotherapy. Asian Pac J Cancer Prev.

[11] Xiao WK, Chen D, Li SQ, Fu SJ, Peng BG, Liang L (2014). Prognostic significance of neutrophil-lymphocyte ratio in hepatocellular carcinoma: a meta-analysis. BMC Cancer.

[12] Inoue D, Ozaka M, Matsuyama M, Yamada I, Takano K, Saiura A (2015). Prognostic value of neutrophil-lymphocyte ratio and level of C-reactive protein in a large cohort of pancreatic cancer patients: a retrospective study in a single institute in Japan. Jpn J Clin Oncol.

[13] Kanda Y (2013). Investigation of the freely available easy-to-use software ‘EZR’ for medical statistics. Bone Marrow Transplant.

[14] Nakamura Y, Nagao J, Saida Y, Watanabe M, Okamoto Y, Asai K (2013). Use of prognostic nutritional index to predict clinical outcomes of patients with terminal stage cancer. Palliative Care Research.

[15] Onodera T, Goseki N, Kosaki G (1984). Prognostic nutritional index in gastrointestinal surgery of malnourished cancer patients. Nippon Geka Gakkai Zasshi.

[16] Zahorec R (2001). Ratio of neutrophil to lymphocyte counts—rapid and simple parameter of systemic inflammation and stress in critically ill. Bratisl Lek Listy.

[17] Teramukai S, Kitano T, Kishida Y, Kawahara M, Kubota K, Komuta K (2009). Pretreatment neutrophil count as an independent prognostic factor in advanced non-small-cell lung cancer: an analysis of Japan Multinational Trial Organisation LC00-03. Eur J Cancer.

[18] Maltoni M, Pirovano M, Nanni O, Marinari M, Indelli M, Gramazio A (1997). Biological indices predictive of survival in 519 Italian terminally ill cancer patients. Italian Multicenter Study Group on Palliative Care. J Pain Symptom Manage.

